# PROX1: A Lineage Tracer for Cortical Interneurons Originating in the Lateral/Caudal Ganglionic Eminence and Preoptic Area

**DOI:** 10.1371/journal.pone.0077339

**Published:** 2013-10-14

**Authors:** Anna Noren Rubin, Nicoletta Kessaris

**Affiliations:** Wolfson Institute for Biomedical Research and Department of Cell and Developmental Biology, University College London, United Kingdom; Instituto de Medicina Molecular, Portugal

## Abstract

The homeobox-encoding gene *Prox1* and its *Drosophila* homologue *prospero* are key regulators of cell fate-specification. In the developing rodent cortex a sparse population of cells thought to correspond to late-generated cortical pyramidal neuron precursors expresses PROX1. Using a series of transgenic mice that mark cell lineages in the subcortical telencephalon and, more specifically, different populations of cortical interneurons, we demonstrate that neurons expressing PROX1 do not represent pyramidal neurons or their precursors but are instead subsets of cortical interneurons. These correspond to interneurons originating in the lateral/caudal ganglionic eminence (LGE/CGE) and a small number of preoptic area (POA)-derived neurons. Expression within the cortex can be detected from late embryonic stages onwards when cortical interneurons are still migrating. There is persistent expression in postmitotic cells in the mature brain mainly in the outer cortical layers. PROX1^+ve^ interneurons express neurochemical markers such as calretinin, neuropeptide Y, reelin and vasoactive intestinal peptide, all of which are enriched in LGE/CGE- and some POA-derived cells. Unlike in the cortex, in the striatum PROX1 marks nearly all interneurons regardless of their origin. Weak expression of PROX1 can also be detected in oligodendrocyte lineage cells throughout the forebrain. Our data show that PROX1 can be used as a genetic lineage tracer of nearly all LGE/CGE- and subsets POA-derived cortical interneurons at all developmental and postnatal stages *in vivo.*

## Introduction

GABAergic interneurons in rodents originate from subpallial regions in the embryonic telencephalon and migrate widely to populate the neocortex, hippocampus, striatum and amygdala [1]–[3]. Genetic lineage tracing has shown that there are three sources of cortical interneurons in the subpallium: the medial ganglionic eminence (MGE), the lateral/caudal ganglionic eminence (LGE/CGE) and the preoptic area (POA) [4]–[11]. Each of these regions generates interneurons with distinct physiological, morphological and molecular characteristics, all of which are further sculpted by local connectivity and network recruitment [1].

Regardless of where they reside within the adult cortex, interneurons that have common origins often share molecular markers, some of which are key regulators of cell identity. For example, the LIM homeobox protein LHX6 is a transcription factor that is expressed in all postmitotic interneurons originating in the MGE. These include parvalbumin (PV)^+^ basket and chandelier cells and somatostatin (SST)^+^ Martinotti and basket cells [12]. Expression of LHX6 is observed throughout development and in mature neurons and loss-of-function studies in mice have demonstrated an essential role for LHX6 in migration and specification of the lineage [13]. SOX6 and the recently reported SATB1 transcriptional modulators have been shown to act downstream of LHX6 conferring maturation and network integration of MGE interneuron subtypes [14]–[17]. These and other markers have not only shed light on the genetics of cortical interneuron development but have also served as invaluable lineage tracers in *in vitro* stem cell differentiation and *in vivo* transplantation studies where differentiated cell types need to be identified [18]–[20].

Interneurons originating in the CGE constitute one third of interneurons in the cortex and hippocampus and include cortical vasoactive intestinal peptide (VIP)^+ve^ bipolar, bitufted and multipolar cells and reelin (RLN)^+ve^SST^−ve^ multipolar cells [8], [9], [21], [22]. Molecular determinants of LGE/CGE-derived interneuron fate remain elusive and as such our knowledge of LGE/CGE interneuron specification and development remains poor. GSX2 is a transcription factor that is expressed throughout the subpallial ventricular zone (VZ) but is particularly enriched in the LGE/CGE and contributes to the specification of bipolar cortical interneurons [23]. The *Nr2f2* gene encoding for the chicken ovalbumin upstream promoter-transcription factor II (COUPTFII) was the first marker to be identified as a factor enriched in - but not restricted to - LGE/CGE-derived interneurons. It functions mainly in directing migration towards a caudal route [24]–[26]. The serotonin receptor HTR3a has been detected in migrating and mature LGE/CGE and POA-derived cortical interneurons but not in MGE-derived ones [21], [22] and SP8 is a transcription factor that marks some LGE/CGE-derived interneurons [27]. The functions of HTR3a and SP8 in cortical interneuron development are unknown.

The homeobox-encoding gene *Prox1* and its *Drosophila* homologue *prospero* have best been described in the developing *Drosophila* nervous system and the vertebrate lymphatic vasculature, where they promote cell fate specification [28], [29]. In the embryonic and postnatal vertebrate nervous system, PROX1 has been detected in subventricular zone (SVZ) where it regulates early stages of neuronal differentiation [30]–[38]. At late embryonic stages and in the postnatal brain there is sparse expression of PROX1 in the cortex [34], [38], [39]. This has been attributed to immature cortical pyramidal cells although their identity has not been confirmed. The scattered distribution of PROX1^+ve^ cells in the cortex is reminiscent of cortical interneurons and prompted us to examine the expression of PROX1 in a series of transgenic mice which label distinct cortical interneuron subsets. We find that PROX1 is not expressed in cortical pyramidal cell precursors. Instead, it identifies LGE/CGE-derived cortical interneurons and a small subset of POA-derived ones at all stages of their development and in the adult cortex, thus acting as a lineage marker for these populations.

## Materials and Methods

### Ethics Statement

All animal work was carried out in accordance with United Kingdom legislation. The protocols have been approved by the UCL Animal Welfare and Ethical Review Board. Postnatal animals were sacrificed by terminal anesthesia using Hypnorm/Hypnovel prior to perfusion fixation. All efforts were made to minimize animal suffering.

### Transgenic Mice

Nkx2.1-Cre [Tg(Nkx2-1-Cre)1Wdr], Lhx6-Cre [Tg(Lhx6-Cre)1Kess], Nkx5.1-Cre, and Dlx1-Venus^fl^ [Tg(Dlx1-Venus)1Kess] transgenic mice and the two reporter mice Rosa26 (R26R)-GFP [Gt(ROSA)26Sor^tm2Sho^] and R26R-YFP [Gt(ROSA)26Sor^tm1(EYFP)Cos^] have been described previously [4], [6], [9], [40], [41]. Mouse colonies were maintained on a mixed C57BL6/CBA background at the Wolfson Institute for Biomedical Research, University College of London.

### Tissue Preparation

The day of the vaginal plug was considered embryonic day (E) 0.5, and the day of birth was considered postnatal day (P) 0. Whole embryo heads (for E12.5) or dissected brains were fixed overnight in 4% (w/v) paraformaldehyde (PFA) in PBS. Postnatal animals were anesthetized prior to perfusion fixation with 4% (w/v) PFA through the left ventricle of the heart. Adult brains were dissected out, sliced into 2 mm slices using a mouse brain coronal matrix and postfixed in 4% PFA overnight. Fixed samples were cryoprotected overnight by immersion in 20% (w/v) sucrose in PBS. All samples were embedded in Tissue-Tek OCT compound (R. A. Lamb Medical Supplies, Eastbourne, UK), frozen on dry ice, and stored at −80°C.

### Immunohystochemistry

Embryonic brains were sectioned coronally to a thickness of 18 µm on a cryostat and collected directly onto Superfrost plus slides (BDH Laboratory Supplies, Poole, UK). Adult sections were cut coronally (30 µm thickness) and were collected in PBS for “free floating” staining. All sections were blocked in PBS containing 10% heat-inactivated sheep serum or fetal calf serum (Sigma, St. Louis, MO) and 0.1% Triton X-100 (Sigma) at room temperature for 1 hr.

Immunohistochemistry was performed with the following primary antibodies: rabbit anti-Prox1 (1∶500; ReliaTech GmbH); goat anti-Prox1 (1∶500; R&D Systems, Inc.); rat anti-GFP IgG2a (1∶1000; Nacalai Tesque, Kyoto,Japan); rabbit anti-calretinin (1∶2000; Swant, Bellizona, Switzerland); sheep anti-neuropeptide Y (1∶500; Abcam); guinea pig anti-vasoactive intestinal peptide (1∶1000; Peninsula Laboratories, San Carlos, CA), mouse anti-parvalbumin (1∶1000; Swant), mouse anti-reelin (1∶2000; kindly provided by A.Goffinet); guinea pig anti-Sox10 (1∶4000; kindly provided by M. Wegner); mouse anti-ki67 (1∶1000, BD Pharmingen). Primary antibodies were diluted in blocking solution and applied overnight at 4°C. Antigen retrieval using citrate buffer (Sigma) was performed for detection of Ki67.

Secondary antibodies AlexaFluor 488-conjugated and AlexaFluor 647-conjugated donkey anti-rabbit IgG, donkey anti-sheep IgG, donkey anti-rat IgG, donkey anti-mouse IgG or goat anti-guinea pig IgG (all used at 1∶750; Invitrogen, Carlsbad, CA) were applied for 1 hr at room temperature. For immunodetection of PROX1, biotin-conjugated donkey anti-rabbit IgG (1∶500; Millipore) or biotin-conjugated donkey anti-goat IgG (1∶200, Jackson Immunochemicals) secondary antibodies were applied for 1 hr at room temperature followed by Avidin/Biotinylated enzyme Complex (ABC) and Tyramide Signal Amplification (TSA) as described previously [42]. Tyramide-Cy3 (Perkin Elmer) was diluted at 1∶100 and the colour was developed for 10 min at room temperature. Floating sections were transferred onto Superfrost plus slides after staining and air dried. All sections were coverslipped with Dako fluorescent mounting medium.

### Image Processing

Images were captured using a Zeiss fluorescent microscope, a Leica confocal microscope or a Perkin Elmer spinning disk confocal miscroscope. Images were processed with Adobe Photoshop CS4 (Adobe Systems Inc., San Jose, CA) for basic level adjustments and montage assembly. Figures were composed in Adobe Illustrator CS4 (Adobe Systems Inc.).

### Quantification

The extent of co-localization between PROX1 and Venus/GFP/YFP or other markers was determined as previously described [4]. In all experiments quantification was carried out in the primary somatosensory cortex between Bregma positions 0.86 and −1.34 mm. Cells were counted in a defined area spanning the pial–white matter extent of the cortex (1250 µm width×30 µm depth). In some cases this was subdivided into 10 equal bins along the dorso-ventral axis and the number of cells in each bin was determined. For all quantification experiments a minimum of three mice at P30 were used. Counts were performed on 2–3 non-consecutive sections from each mouse. Results are expressed as mean ± standard error of the mean (SEM). Graphical representations of the data and statistical analyses were performed using GraphPad Prism 6 (GraphPad Software, Inc., San Diego, CA).

## Results

### Expression of PROX1 in the Ganglionic Eminences of the Developing Telencephalon

**Figure 1:**
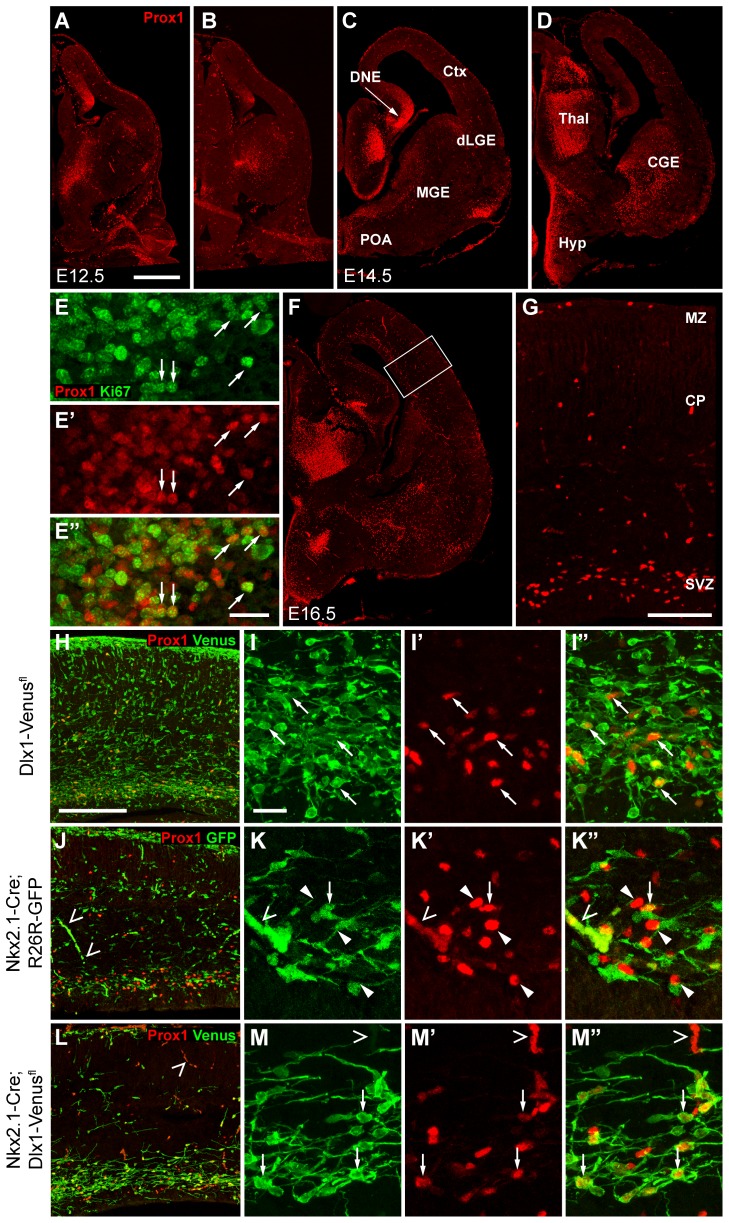
PROX1 expression in the embryonic forebrain. (**A–D**) Immunolabeling for PROX1 in coronal sections of the forebrain at E12.5 and E14.5. Expression can be seen in the SVZ of the MGE, dorsal LGE (dLGE), CGE and POA as well as in the dentate neuroepithelium (DNE), thalamus (Thal) and hypothalamus (Hyp). (**E**) Co-localization between PROX1 and Ki67 in the SVZ of the MGE at E14.5. (**F–G**) At E16.5, sparse PROX1^+ve^ cells are visible in the cortex (shown at higher magnification in **G**). (**H–M**) Immunolabeling for PROX1 and Venus/GFP in transgenic mouse lines that label either all cortical interneurons (**H,I**), MGE/POA-derived ones (**J,K**) or LGE/CGE–derived ones (**L,M**). PROX1^+ve^ cells in the cortex at E16.5 correspond to migrating immature interneurons expressing Venus (**H, I**). MGE/POA-derived migrating interneurons are largely immunonegative for PROX1 (**J, K**). Most PROX1^+ve^ cells in the cortex represent LGE/CGE-derived migrating interneurons (**L, M**). Arrows indicate PROX1^+ve^ Venus/GFP^+ve^ interneurons, arrowheads indicate PROX1^+ve^ Venus/GFP-negative cells. Open arrowheads point to autofluorescence from red blood cells. Scale bars: **A–D, F**, 500 µm; **E**, 25 µm; **G**, 100 µm; **H**, **J**, **L**, 100 µm; **I**, **K**, **M**, 20 µm.

Previous studies had shown expression of PROX1 in the SVZ of the subpallial telencephalon [34], [38]. We examined this more closely to determine whether expression coincides with the three germinal regions where cortical interneurons are generated. At E12.5, E14.5 and E16.5 PROX1 could be detected in the SVZ of the MGE, the LGE/CGE and the POA (Fig. 1A–F). There was no co-localization between PROX1 and the M-phase marker PH3 (data not shown), as previously reported [34], [38], but there was extensive co-expression with the general proliferation marker Ki67 in the SVZ of the ganglionic eminences (Fig. 1E). At E16.5 cells expressing PROX1 could also be detected in the cortex (Fig. 1F,G). These were few in number and were located mainly within the SVZ and largely absent from the cortical plate (CP) and marginal zone (MZ) (Fig. 1G). The SVZ is one of the main tangential migratory routes of cortical interneurons [43], [44], suggesting that PROX1-expressing cells in the developing cortex may correspond to immature migrating cortical interneurons.

### Migrating Immature LGE/CGE-derived Cortical Interneurons Express PROX1

To determine whether PROX1-expressing cells in the embryonic cortex represent migrating interneurons we made use of *Dlx1-Venus^fl^* mice, which express the fluorescent protein Venus in all cortical GABAergic interneurons [9]. All PROX1^+ve^ cells in the cortex co-expressed Venus confirming that they are indeed interneurons (Fig. 1H,I). As interneurons in the cortex can be generated in one of three areas (MGE, LGE/CGE and POA), we used transgenic mouse lines which allowed us to fate-map cell lineages generated in these areas and identify interneurons derived from them. These were *Nkx2.1-Cre;R26R-GFP* which label MGE- and POA-derived cells [4], and *Nkx2.1-Cre;Dlx1-Venus^fl^* mice, which label LGE/CGE-derived cells (*Nkx2.1-Cre* subtracts the Venus label from MGE and POA cell derivatives) [9]. There was only occasional co-localization between GFP and PROX1 in *Nkx2.1-Cre;R26R-GFP* embryos (Fig. 1J,K) whereas nearly all PROX1^+ve^ cells co-expressed Venus in *Nkx2.1-Cre;Dlx1-Venus^fl^* mice (Fig. 1L,M). Our data suggest that PROX1 is expressed in LGE/CGE-derived interneurons but largely absent from MGE/POA-derived ones, raising the possibility that PROX1 may be a lineage marker for this population.

### PROX1 Labels LGE/CGE- and POA-derived Interneurons in the Adult Cortex

**Figure 2:**
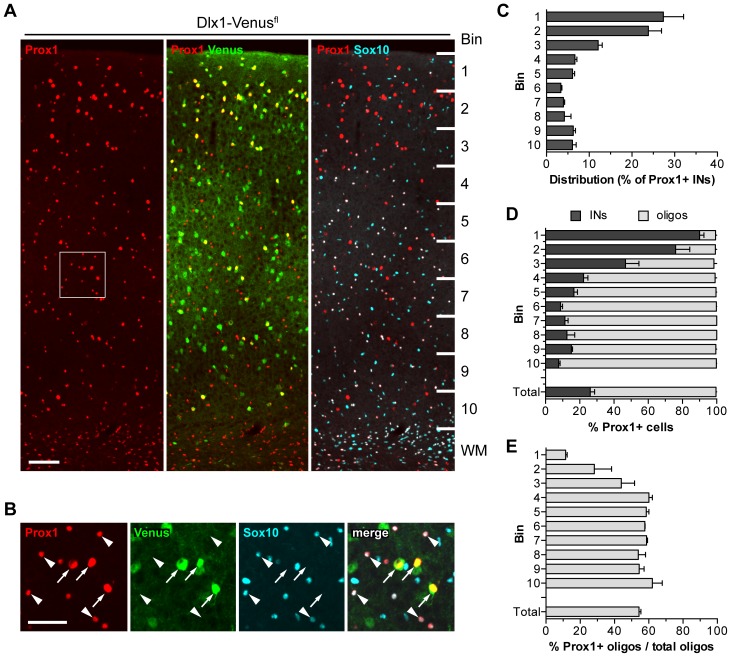
PROX1 is expressed in cortical interneurons and oligodendrocyte lineage cells at P30. (**A**) PROX1, Venus and SOX10 expression in the primary somatosensory cortex of the *Dlx1-Venus^fl^* mouse. The cortex is divided into 10 equal bins for quantification. (**B**) Higher magnification of the boxed area in **A**. Nuclear PROX1 expression is evident in interneurons (arrows) and oligodendrocyte lineage cells (arrowheads) throughout all the layers of the cortex. Interneurons generally showed more intense labelling for PROX1. (**C**) Distribution of PROX1^+ve^ Venus^+ve^ interneurons quantified across cortical bins. (**D**) Quantification of the percentage of PROX1^+ve^ cells expressing Venus (interneurons or INs) or SOX10 (oligos) in each bin. (**E**) Contribution of PROX1^+ve^ cells to the total oligodendrocyte lineage population. WM, white matter. Scale bars: **A**, 100 µm; **B**, 50 µm.

The data so far indicated that PROX1 is expressed in immature migrating interneurons originating in the LGE/CGE at embryonic stages. To determine whether this expression is maintained at later stages we examined cortices from adult transgenic mice. Cells co-expressing Venus and high levels of PROX1 were found in *Dlx1-Venus^fl^* mice suggesting that expression is maintained in mature interneuron subsets (Fig. 2A–C). These cells were located mainly in the outer layers of the cortex (Fig. 2C). In addition to interneurons, an abundant population of cells expressing lower levels of PROX1 was observed in the middle and lower layers of the cortex and in the white matter (Fig. 2A). PROX1^+ve^Venus^−ve^ cells in the cortex and white matter co-expressed the oligodendrocyte lineage marker SOX10 [45], indicating that they represent subsets of oligodendrocyte precursors and/or mature myelinating oligodendrocytes (Fig. 2A,B,D,E). This expression in oligodendrocytes was observed with both anti-PROX1 antibodies used in this study and was confirmed by *in situ* hybridization for *Prox1* in the adult brain (data not shown). Quantification of co-localization of PROX1, SOX10 and Venus indicated that interneurons and oligodendrocytes account for the entire population of PROX1^+ve^ cells in all layers of the cortex (Fig. 2D).

**Figure 3:**
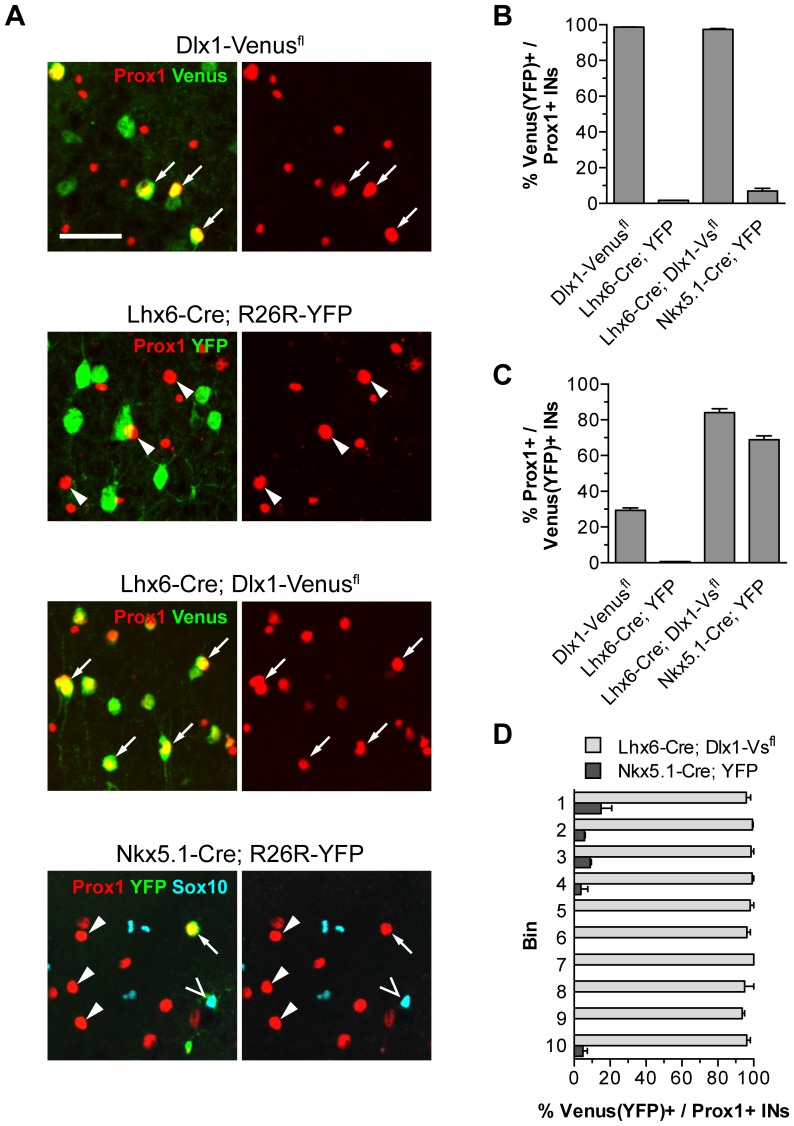
PROX1 labels cortical interneurons derived from the LGE/CGE and POA. (**A**) Co-expression of PROX1 and Venus/YFP in transgenic mouse lines that label interneurons derived from different progenitor regions. Arrows indicate co-expression, arrowheads indicate Venus/YFP-negative PROX1^+ve^ cells. Open arrowheads indicate YFP-labeled oligodendrocytes. (**B**) Contribution of the different progenitor regions to the PROX1^+ve^ interneuron population. (**C**) Co-localization of PROX1 and Venus/YFP expressed as a percentage of the total interneurons derived from each progenitor region. (**D**) The contribution of LGE/CGE/POA (light grey) or POA only (dark grey) to the PROX1^+ve^ population in each bin. The POA contributes mainly upper layer PROX1^+ve^ interneurons. Scale bar: **A**, 50 µm.

The distribution of interneurons expressing PROX1 in the outer layers of the cortex is reminiscent of LGE/CGE and some POA-derived populations [6], [8], [9]. To determine whether PROX1 is indeed a lineage marker for these cells we examined and quantified the extent of co-localization between Venus/YFP and PROX1 in our transgenic mice. In *Dlx1-Venus^fl^* mice 99% of PROX1^+ve^SOX10^−ve^ cells in the somatosensory cortex co-expressed Venus and represented ∼30% of the total interneuron population (Fig. 3A–C). As *Nkx2.1-Cre* fails to recombine in the dorsal MGE, for quantification purposes in the adult brain we switched to *Lhx6-Cre* which labels all MGE-derived interneurons in the cortex [4]. Unlike *Nkx2.1-Cre*, *Lhx6-Cre* does not recombine in the POA. There was almost no co-localization between PROX1 and YFP in *Lhx6-Cre;R26R-YFP* transgenic mice whereas 97% of PROX1^+ve^SOX10^−ve^ cells co-expressed Venus in *Lhx6-Cre;Dlx1-Venus^fl^* mice (Fig. 3A–C). These data indicate that PROX1^+ve^ interneurons are derived mainly from the LGE/CGE and possibly the POA. To directly visualize POA-derived cells we made use of *Nkx5.1-Cre;R26R-YFP* transgenic mice, which express YFP in a subset of POA-derived interneurons [6]. 69% of YFP^+ve^SOX10^−ve^ interneurons co-expressed PROX1 in these mice and represented <10% of the total PROX1^+ve^ interneuron population (Fig. 3A–C). *Nkx5.1*-derived POA interneurons expressing PROX1 were located almost exclusively within the outer cortical layers (Fig. 3D). Altogether our findings show that PROX1 expression is confined to LGE/CGE and POA-derived cortical interneurons but is excluded from MGE-derived populations.

### PROX1 Identifies CR- NPY- RLN- and VIP-expressing Interneurons in the Adult Cortex

**Figure 4:**
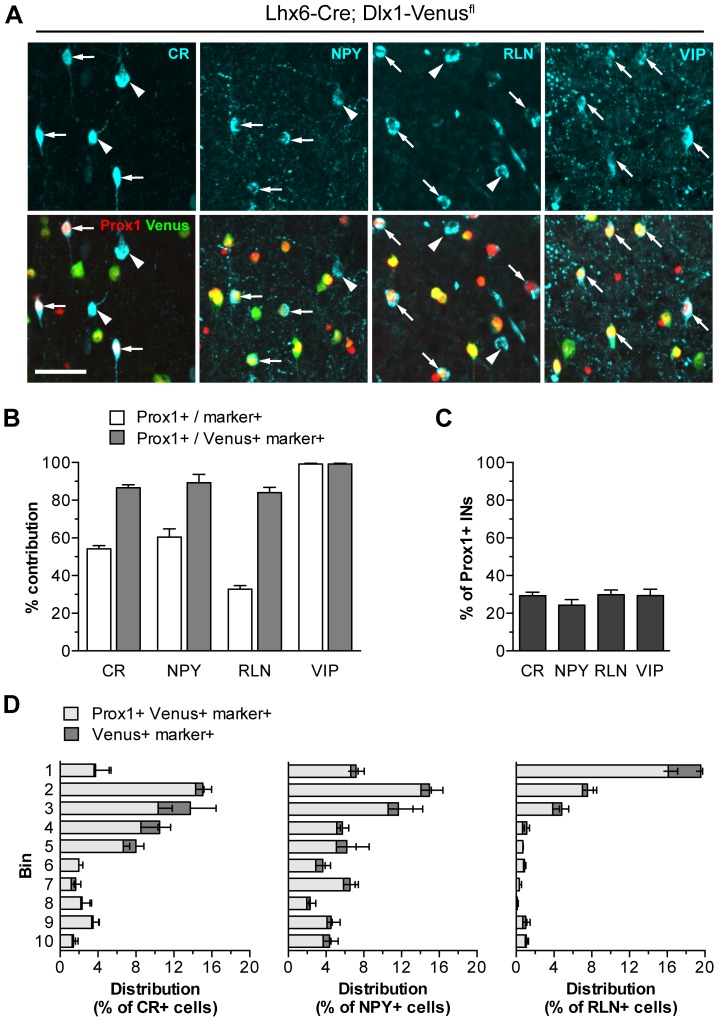
Co-expression of PROX1 with cortical interneuron markers. (**A**) PROX1 is expressed in CR, NPY, RLN and VIP cortical interneurons. Arrows indicate PROX1^+ve^ marker^+ve^ cells, arrowheads indicate PROX1^−ve^ cells. (**B**) Quantification of the contribution of PROX1^+ve^ cells to each of the marker^+ve^ populations (white), and specifically to the LGE/CGE/POA-derived populations labelled with Venus (grey) in the *Lhx6-Cre;Dlx1-Venus^fl^* mouse. (**C**) The percentage of PROX1^+ve^ interneurons co-expressing each of the interneuron markers. (**D**) Laminar distribution of the interneurons co-expressing PROX1 and CR, NPY or RLN (light grey) as well as the total LGE/CGE/POA-derived marker^+ve^ populations (dark grey). Scale bar: **A**, 50 µm.

Data so far showed that PROX1 is expressed in 84% of all LGE/CGE/POA-derived cortical interneurons (Fig. 3C). To determine whether these PROX1^+ve^ cells correspond to particular subtypes we characterized their neurochemical profile using markers that define LGE/CGE and POA interneuron subsets. PROX1 was found to be expressed in large numbers of calretinin (CR)-, neuropeptide Y (NPY)- and RLN-positive cells as well as in 100% of the VIP-expressing population (Fig. 4A,B). As subsets of CR-, NPY- and RLN-positive cells are also generated in the MGE, we quantified the co-localization of PROX1 with the different markers specifically in LGE/CGE/POA-lineage tracing mice. >80% of Venus^+ve^ cells expressing CR, NPY, or RLN showed co-expression of PROX1 in *Lhx6-Cre;Dlx1-Venus^fl^* mice (Fig. 4B). Each population represented 25–30% of PROX1^+ve^ interneurons in the somatosensory cortex (Fig. 4C). These PROX1^+ve^Venus^+ve^ cells were located mainly in the outer layers of the cortex where most LGE/CGE- and POA-derived interneurons reside (Fig. 4D). Our data indicate that PROX1 is expressed in all the different interneuron subtypes known to originate in the LGE/CGE.

### PROX1 Expression in Hippocampal Interneurons

**Figure 5:**
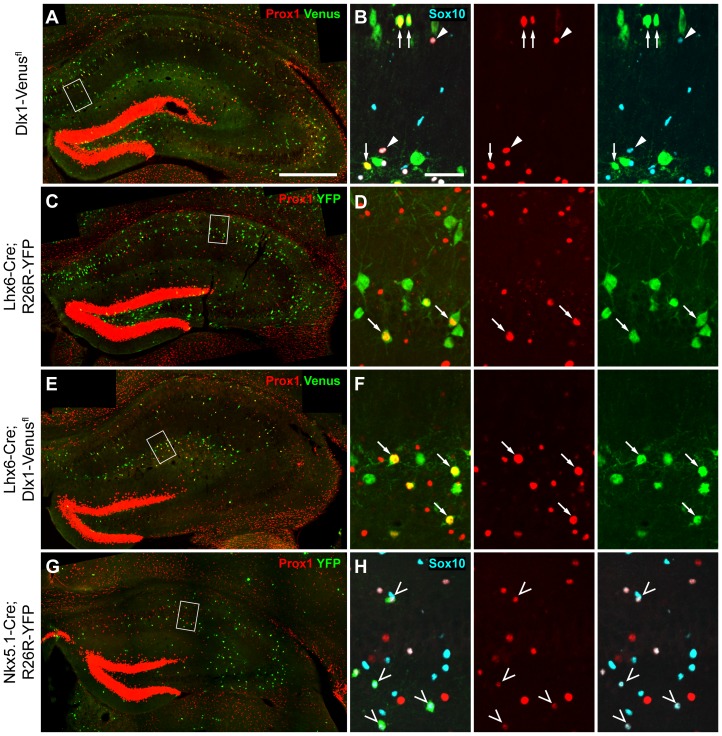
Expression of PROX1 in the hippocampus at P30. (**A, C, E, G**) PROX1 co-expression with Venus/YFP in the hippocampus of the different transgenic mouse lines. (**B, D, F, G**) Higher magnification images of the boxed areas. Arrows indicate PROX1^+ve^ Venus/YFP^+ve^ interneurons, arrowheads indicate PROX1^+ve^SOX10^+ve^ oligodendrocytes, open arrowheads indicate YFP^+ve^ oligodendrocytes in *Nkx5.1-Cre;R26R-YFP* transgenic mice. Scale bars: **A**, **C**, **E**, **G**, 500 µm; **B**, **D**, **F**, **H**, 50 µm.

Interneurons in the hippocampus and cortex have common origins in the ganglionic eminences and POA and to date there have been no lineage tracing markers that distinguish between the two populations. To determine whether PROX1 is marking the same lineages in the hippocampus as it does in the cortex we examined its expression in our four transgenic mouse lines. As expected, there was expression of PROX1 in some Venus^+ve^ cells in *Dlx1-Venus^fl^* mice in all layers and regions of the hippocampus (Fig. 5A,B). The majority of YFP-expressing interneurons in the hippocampus of *Lhx6-Cre;R26R-YFP* mice did not express PROX1 although greater co-localization was observed in these mice compared to the cortex (Fig. 5C,D). On the other hand, the vast majority of Venus^+ve^ cells co-expressed PROX1 in *Lhx6-Cre;Dlx1-Venus^fl^* mice (Fig. 5E,F). Very little or no expression of PROX1 could be detected in *Nkx5.1-Cre;R26R-YFP* mice. Most YFP-expressing cells present in these animals represent oligodendrocyte lineage cells expressing SOX10 and low levels of PROX1 (Fig. 5G,H). These data are consistent with PROX1 being expressed in most LGE/CGE-derived interneurons of the hippocampus. The *Lhx6-Cre;R26R-YFP*-labeled interneurons expressing PROX1 in the hippocampus may represent a small proportion of MGE-derived interneurons or a subset of the POA-derived interneurons that express LHX6 [5], [6].

### Expression of PROX1 in Striatal Interneurons is Independent of Origin

**Figure 6:**
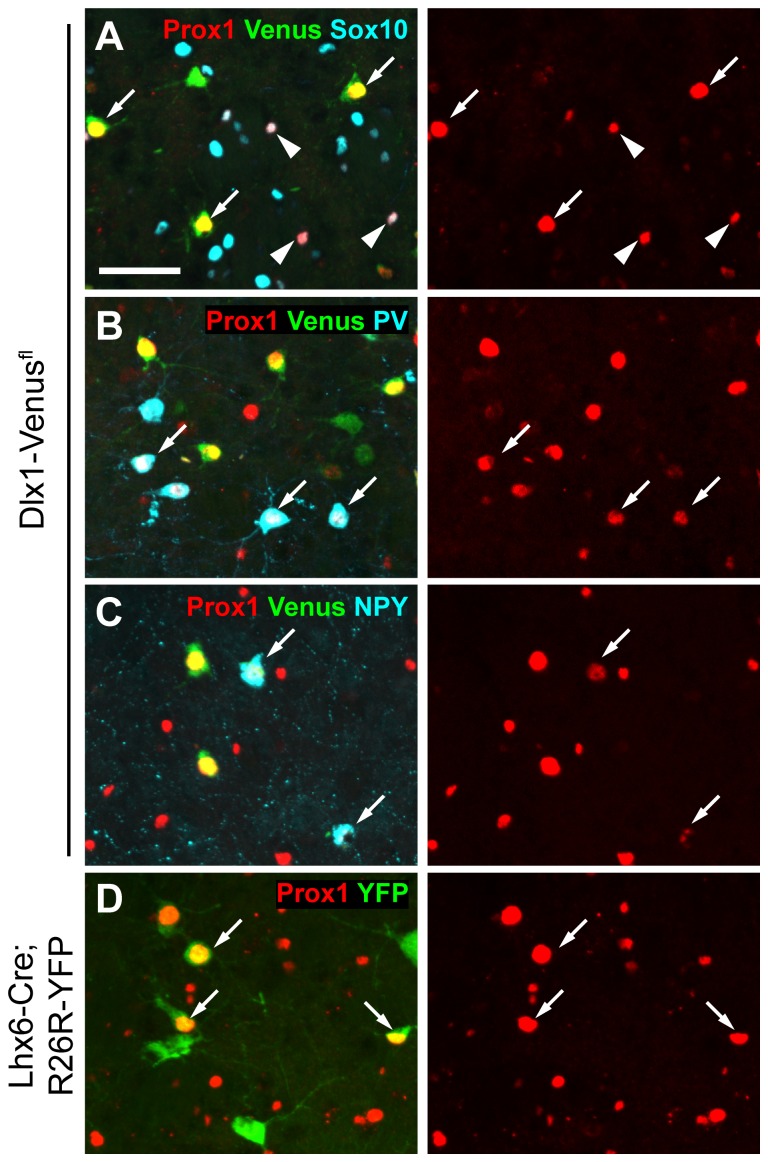
Expression of PROX1 in the striatum at P30. (**A**) PROX1 labels most Venus^+ve^ striatal interneurons (arrows) in the *Dlx1-Venus^fl^* mouse, and some oligodendrocyte lineage cells (arrowheads). PROX1^+ve^ interneurons include the MGE-derived subtypes expressing PV (**B**) and NPY (**C**). (**D**) PROX1 co-localizes with MGE-derived striatal interneurons expressing YFP in the *Lhx6-Cre;R26R-YFP* mouse. Scale bar: **A–D**, 50 µm.

PROX1 expression is also maintained into adulthood in scattered cells of the striatum. We examined this expression in our transgenic mice to determine whether this transcription factor shows lineage specificity in telencephalic regions other than the cortex and hippocampus. We analyzed expression in *Dlx1-Venus^fl^* mice which label nearly all striatal interneurons (our unpublished observations) and *Lhx6-Cre;R26R-YFP* mice which label only MGE-derived populations [46]. PROX1-expressing cells in the striatum represented both interneurons and oligodendrocyte lineage cells as shown by co-expression with either Venus or SOX10 in *Dlx1-Venus^fl^* mice (Fig. 6A). Surprisingly, nearly all PV^+ve^ and NPY^+ve^ interneurons of the striatum co-expressed PROX1 even though these originate in the MGE (Fig. 6B,C) [46]. Indeed, there was extensive co-localization between PROX1 and YFP in *Lhx6-Cre;R26R-YFP* transgenic mice indicating that most MGE-derived striatal interneurons express PROX1 (Fig. 6D). Altogether the data suggest that PROX1 can be used to identify LGE/CGE/POA-derived interneurons in the cortex but in the striatum it has a broader expression pattern and labels the majority of interneurons regardless of their origin.

## Discussion

Our data show that PROX1 is a lineage marker for LGE/CGE- and a subset of POA-derived cortical interneurons. It can be used to identify these cells from the initial stages of migration into the cortex as well as in the mature brain. During early development PROX1 expression is observed in the SVZ of all three ganglionic eminences as well as the POA, which suggests that the gene may be actively down-regulated in MGE interneurons migrating to the cortex. In the striatum however, expression of PROX1 in interneurons is independent of origin and can be detected in nearly all subtypes.

What could be the function of PROX1 in the telencephalic SVZ? One of the functions of *prospero* and the vertebrate PROX1 is to regulate the balance between cycling and differentiation of precursors [47], [48]. For example, in the chick spinal cord, there is PROX1 expression in the interface between the VZ and the mantle where it is thought to act as a repressor of *Notch1* expression allowing cell cycle exit and initiation of neuronal differentiation [32]. Its expression in Ki67^+ve^ cells in the SVZ of the telencephalic ganglionic eminences suggests that it may have a similar role in the regulation of cell cycle exit and differentiation not only of cortical and striatal interneurons but also of other neuronal populations which are generated from the ganglionic eminences, including striatal and pallidal projection neurons.

In the dentate gyrus of the embryonic and adult hippocampus, PROX1 is activated downstream of Wnt signals and is required for maintenance of intermediate precursors [33], [35]. In these cells PROX1 also acts as a postmitotic cell fate determinant specifying granule cell identity over CA3 pyramidal cell fate [31] and later on it is required for maturation and survival of immature granule cells [35]. In the lymphatic system PROX1 expression is maintained in lymphatic endothelial cells (LEC) and is required throughout life for maintenance of cell identity [49]. Whether it has similar roles in the developing telencephalon remains to be determined. The constitutive expression of PROX1 in LGE/CGE/POA-derived cortical interneurons and all interneurons of the striatum suggests that it may act as a fate-selector gene for these lineages over alternative fates at early stages and later it may promote maturation, survival and/or maintenance of interneuron identity.

The expression of PROX1 in dividing precursors, immature migrating interneurons as well as mature fully integrated cells suggests multiple roles at different developmental stages. The mechanism by which one single transcription factor may exert multiple functions is unknown but may involve differential binding partners and/or distinct downstream targets. In the developing lymphatic vasculature PROX1 is essential for the initial steps of LEC specification from developing veins [28]. Recent evidence has shown that COUPTFII acts as an upstream activator and a binding partner of PROX1 during LEC specification [28], [50]. This genetic and physical interaction between COUPTFII and PROX1 may be conserved also in the telencephalon where the two genes are co-expressed in the ganglionic eminences and in migrating LGE/CGE/POA-derived cortical interneurons [24]–[26]. A possible association with COUPTFII may implicate PROX1 in caudal migration. The function of PROX1 and its relationship to COUPTFII and SP8 in migrating and mature LGE/CGE/POA-derived interneurons awaits further investigation.

The use of markers for identifying cell lineages is an incredibly powerful tool, not only for investigating the normal development of these cells *in vivo* but also in stem cell differentiation and transplantation studies. For example, NKX2.1 and LHX6, two transcription factors that define MGE lineages, have been used to identify, isolate and tag prospective MGE cortical interneuron precursors from mouse and human embryonic stem cells during *in vitro* differentiation and following *in vivo* transplantation [18]–[20]. Such studies are lacking for LGE/CGE-derived cortical interneurons partly because of the paucity of definitive lineage markers. With several studies in mice showing that transplanted embryonic precursors can ameliorate symptoms of seizure-associated disorders such as epilepsy, the identification of lineage-specific genes and mature interneuron subtype markers is now of clinical importance as well [51]–[53]. Our data show that PROX1 can be added to the genetic repertoire of LGE/CGE/POA-derived cortical interneurons and can be used to identify these cells at all stages of their embryonic and adult life.

## References

[1] Fishell G, Rudy B (2011) Mechanisms of inhibition within the telencephalon: “where the wild things are”. Annu Rev Neurosci 34: 535–567. 21469958 10.1146/annurev-neuro-061010-113717 PMC3556485

[2] Gelman DM, Marin O (2010) Generation of interneuron diversity in the mouse cerebral cortex. Eur J Neurosci 31: 2136–2141. 20529125 10.1111/j.1460-9568.2010.07267.x

[3] Welagen J, Anderson S (2011) Origins of neocortical interneurons in mice. Dev Neurobiol 71: 10–17. 21154906 10.1002/dneu.20857

[4] Fogarty M, Grist M, Gelman D, Marin O, Pachnis V, et al (2007) Spatial genetic patterning of the embryonic neuroepithelium generates GABAergic interneuron diversity in the adult cortex. J Neurosci 27: 10935–10946. 17928435 10.1523/JNEUROSCI.1629-07.2007 PMC6672847

[5] Gelman D, Griveau A, Dehorter N, Teissier A, Varela C, et al (2011) A wide diversity of cortical GABAergic interneurons derives from the embryonic preoptic area. J Neurosci 31: 16570–16580. 22090484 10.1523/JNEUROSCI.4068-11.2011 PMC6633309

[6] Gelman DM, Martini FJ, Nobrega-Pereira S, Pierani A, Kessaris N, et al (2009) The embryonic preoptic area is a novel source of cortical GABAergic interneurons. J Neurosci 29: 9380–9389. 19625528 10.1523/JNEUROSCI.0604-09.2009 PMC6665570

[7] Miyoshi G, Butt SJ, Takebayashi H, Fishell G (2007) Physiologically distinct temporal cohorts of cortical interneurons arise from telencephalic Olig2-expressing precursors. J Neurosci 27: 7786–7798. 17634372 10.1523/JNEUROSCI.1807-07.2007 PMC6672881

[8] Miyoshi G, Hjerling-Leffler J, Karayannis T, Sousa VH, Butt SJ, et al (2010) Genetic fate mapping reveals that the caudal ganglionic eminence produces a large and diverse population of superficial cortical interneurons. J Neurosci 30: 1582–1594. 20130169 10.1523/JNEUROSCI.4515-09.2010 PMC2826846

[9] Rubin AN, Alfonsi F, Humphreys MP, Choi CK, Rocha SF, et al (2010) The germinal zones of the basal ganglia but not the septum generate GABAergic interneurons for the cortex. J Neurosci 30: 12050–12062. 20826668 10.1523/JNEUROSCI.6178-09.2010 PMC3044873

[10] Sousa VH, Miyoshi G, Hjerling-Leffler J, Karayannis T, Fishell G (2009) Characterization of Nkx6–2-derived neocortical interneuron lineages. Cereb Cortex 19 Suppl 1i1–10. 19363146 10.1093/cercor/bhp038 PMC2693535

[11] Xu Q, Tam M, Anderson SA (2008) Fate mapping Nkx2.1-lineage cells in the mouse telencephalon. J Comp Neurol 506: 16–29. 17990269 10.1002/cne.21529

[12] Anastasiades PG, Butt SJ (2011) Decoding the transcriptional basis for GABAergic interneuron diversity in the mouse neocortex. Eur J Neurosci 34: 1542–1552. 22103412 10.1111/j.1460-9568.2011.07904.x

[13] Liodis P, Denaxa M, Grigoriou M, Akufo-Addo C, Yanagawa Y, et al (2007) Lhx6 activity is required for the normal migration and specification of cortical interneuron subtypes. J Neurosci 27: 3078–3089. 17376969 10.1523/JNEUROSCI.3055-06.2007 PMC6672459

[14] Azim E, Jabaudon D, Fame RM, Macklis JD (2009) SOX6 controls dorsal progenitor identity and interneuron diversity during neocortical development. Nat Neurosci 12: 1238–1247. 19657336 10.1038/nn.2387 PMC2903203

[15] Batista-Brito R, Rossignol E, Hjerling-Leffler J, Denaxa M, Wegner M, et al (2009) The cell-intrinsic requirement of Sox6 for cortical interneuron development. Neuron 63: 466–481. 19709629 10.1016/j.neuron.2009.08.005 PMC2773208

[16] Close J, Xu H, De Marco GN, Batista-Brito R, Rossignol E, et al (2012) Satb1 is an activity-modulated transcription factor required for the terminal differentiation and connectivity of medial ganglionic eminence-derived cortical interneurons. J Neurosci 32: 17690–17705. 23223290 10.1523/JNEUROSCI.3583-12.2012 PMC3654406

[17] Denaxa M, Kalaitzidou M, Garefalaki A, Achimastou A, Lasrado R, et al (2012) Maturation-promoting activity of SATB1 in MGE-derived cortical interneurons. Cell Rep 2: 1351–1362. 23142661 10.1016/j.celrep.2012.10.003 PMC3607226

[18] Cambray S, Arber C, Little G, Dougalis AG, de P, V, Ungless MA, et al (2012) Activin induces cortical interneuron identity and differentiation in embryonic stem cell-derived telencephalic neural precursors. Nat Commun 3: 841. 22588303 10.1038/ncomms1817

[19] Maroof AM, Brown K, Shi SH, Studer L, Anderson SA (2010) Prospective isolation of cortical interneuron precursors from mouse embryonic stem cells. J Neurosci 30: 4667–4675. 20357117 10.1523/JNEUROSCI.4255-09.2010 PMC2868507

[20] Maroof AM, Keros S, Tyson JA, Ying SW, Ganat YM, et al (2013) Directed differentiation and functional maturation of cortical interneurons from human embryonic stem cells. Cell Stem Cell 12: 559–572. 23642365 10.1016/j.stem.2013.04.008 PMC3681523

[21] Lee S, Hjerling-Leffler J, Zagha E, Fishell G, Rudy B (2010) The largest group of superficial neocortical GABAergic interneurons expresses ionotropic serotonin receptors. J Neurosci 30: 16796–16808. 21159951 10.1523/JNEUROSCI.1869-10.2010 PMC3025500

[22] Vucurovic K, Gallopin T, Ferezou I, Rancillac A, Chameau P, et al (2010) Serotonin 3A Receptor Subtype as an Early and Protracted Marker of Cortical Interneuron Subpopulations. Cereb Cortex 20: 2333–2347. 20083553 10.1093/cercor/bhp310 PMC2936799

[23] Xu Q, Guo L, Moore H, Waclaw RR, Campbell K, et al (2010) Sonic hedgehog signaling confers ventral telencephalic progenitors with distinct cortical interneuron fates. Neuron 65: 328–340. 20159447 10.1016/j.neuron.2010.01.004 PMC2868511

[24] Cai Y, Zhang Q, Wang C, Zhang Y, Ma T, et al (2013) Nuclear receptor COUP-TFII-expressing neocortical interneurons are derived from the medial and lateral/caudal ganglionic eminence and define specific subsets of mature interneurons. J Comp Neurol 521: 479–497. 22791192 10.1002/cne.23186

[25] Kanatani S, Yozu M, Tabata H, Nakajima K (2008) COUP-TFII is preferentially expressed in the caudal ganglionic eminence and is involved in the caudal migratory stream. J Neurosci 28: 13582–13591. 19074032 10.1523/JNEUROSCI.2132-08.2008 PMC6671763

[26] Yozu M, Tabata H, Nakajima K (2005) The caudal migratory stream: a novel migratory stream of interneurons derived from the caudal ganglionic eminence in the developing mouse forebrain. J Neurosci 25: 7268–7277. 16079409 10.1523/JNEUROSCI.2072-05.2005 PMC6725225

[27] Ma T, Zhang Q, Cai Y, You Y, Rubenstein JL, et al (2012) A subpopulation of dorsal lateral/caudal ganglionic eminence-derived neocortical interneurons expresses the transcription factor Sp8. Cereb Cortex 22: 2120–2130. 22021915 10.1093/cercor/bhr296

[28] Francois M, Harvey NL, Hogan BM (2011) The transcriptional control of lymphatic vascular development. Physiology (Bethesda ) 26: 146–155. 21670161 10.1152/physiol.00053.2010

[29] Fuerstenberg S, Broadus J, Doe CQ (1998) Asymmetry and cell fate in the Drosophila embryonic CNS. Int J Dev Biol 42: 379–383. 9654022

[30] Galeeva A, Treuter E, Tomarev S, Pelto-Huikko M (2007) A prospero-related homeobox gene Prox-1 is expressed during postnatal brain development as well as in the adult rodent brain. Neuroscience 146: 604–616. 17368742 10.1016/j.neuroscience.2007.02.002

[31] Iwano T, Masuda A, Kiyonari H, Enomoto H, Matsuzaki F (2012) Prox1 postmitotically defines dentate gyrus cells by specifying granule cell identity over CA3 pyramidal cell fate in the hippocampus. Development 139: 3051–3062. 22791897 10.1242/dev.080002

[32] Kaltezioti V, Kouroupi G, Oikonomaki M, Mantouvalou E, Stergiopoulos A, et al (2010) Prox1 regulates the notch1-mediated inhibition of neurogenesis. PLoS Biol 8: e1000565. 21203589 10.1371/journal.pbio.1000565 PMC3006385

[33] Karalay O, Doberauer K, Vadodaria KC, Knobloch M, Berti L, et al (2011) Prospero-related homeobox 1 gene (Prox1) is regulated by canonical Wnt signaling and has a stage-specific role in adult hippocampal neurogenesis. Proc Natl Acad Sci U S A 108: 5807–5812. 21436036 10.1073/pnas.1013456108 PMC3078392

[34] Lavado A, Oliver G (2007) Prox1 expression patterns in the developing and adult murine brain. Dev Dyn 236: 518–524. 17117441 10.1002/dvdy.21024

[35] Lavado A, Lagutin OV, Chow LM, Baker SJ, Oliver G (2010) Prox1 is required for granule cell maturation and intermediate progenitor maintenance during brain neurogenesis. PLoS Biol 8 e1000460. 10.1371/journal.pbio.1000460 PMC2923090 20808958

[36] Long JE, Cobos I, Potter GB, Rubenstein JL (2009) Dlx1&2 and Mash1 transcription factors control MGE and CGE patterning and differentiation through parallel and overlapping pathways. Cereb Cortex 19 Suppl 1i96–106. 19386638 10.1093/cercor/bhp045 PMC2693539

[37] Oliver G, Sosa-Pineda B, Geisendorf S, Spana EP, Doe CQ, et al (1993) Prox1, a prospero-related homeobox gene expressed during mouse development. Mech Dev 44: 3–16. 7908825 10.1016/0925-4773(93)90012-m

[38] Torii M, Matsuzaki F, Osumi N, Kaibuchi K, Nakamura S, et al (1999) Transcription factors Mash-1 and Prox-1 delineate early steps in differentiation of neural stem cells in the developing central nervous system. Development 126: 443–456. 9876174 10.1242/dev.126.3.443

[39] Elkouris M, Balaskas N, Poulou M, Politis PK, Panayiotou E, et al (2011) Sox1 maintains the undifferentiated state of cortical neural progenitor cells via the suppression of Prox1-mediated cell cycle exit and neurogenesis. Stem Cells 29: 89–98. 21280160 10.1002/stem.554

[40] Mao X, Fujiwara Y, Chapdelaine A, Yang H, Orkin SH (2001) Activation of EGFP expression by Cre-mediated excision in a new ROSA26 reporter mouse strain. Blood 97: 324–326. 11133778 10.1182/blood.v97.1.324

[41] Srinivas S, Watanabe T, Lin CS, William CM, Tanabe Y, et al (2001) Cre reporter strains produced by targeted insertion of EYFP and ECFP into the ROSA26 locus. BMC Dev Biol 1: 4. 11299042 10.1186/1471-213X-1-4 PMC31338

[42] Magno L, Oliveira MG, Mucha M, Rubin AN, Kessaris N (2012) Multiple embryonic origins of nitric oxide synthase-expressing GABAergic neurons of the neocortex. Front Neural Circuits 6: 65. 23015780 10.3389/fncir.2012.00065 PMC3449337

[43] Lavdas AA, Grigoriou M, Pachnis V, Parnavelas JG (1999) The medial ganglionic eminence gives rise to a population of early neurons in the developing cerebral cortex. J Neurosci 19: 7881–7888. 10479690 10.1523/JNEUROSCI.19-18-07881.1999 PMC6782477

[44] Wichterle H, Turnbull DH, Nery S, Fishell G, Alvarez-Buylla A (2001) In utero fate mapping reveals distinct migratory pathways and fates of neurons born in the mammalian basal forebrain. Development 128: 3759–3771. 11585802 10.1242/dev.128.19.3759

[45] Stolt CC, Rehberg S, Ader M, Lommes P, Riethmacher D, et al (2002) Terminal differentiation of myelin-forming oligodendrocytes depends on the transcription factor Sox10. Genes Dev 16: 165–170. 11799060 10.1101/gad.215802 PMC155320

[46] Fragkouli A, van Wijk NV, Lopes R, Kessaris N, Pachnis V (2009) LIM homeodomain transcription factor-dependent specification of bipotential MGE progenitors into cholinergic and GABAergic striatal interneurons. Development 136: 3841–3851. 19855026 10.1242/dev.038083 PMC2766344

[47] Choksi SP, Southall TD, Bossing T, Edoff K, de WE, Fischer BE, et al (2006) Prospero acts as a binary switch between self-renewal and differentiation in Drosophila neural stem cells. Dev Cell 11: 775–789. 17141154 10.1016/j.devcel.2006.09.015

[48] Li L, Vaessin H (2000) Pan-neural Prospero terminates cell proliferation during Drosophila neurogenesis. Genes Dev 14: 147–151. 10652268 PMC316344

[49] Johnson NC, Dillard ME, Baluk P, McDonald DM, Harvey NL, et al (2008) Lymphatic endothelial cell identity is reversible and its maintenance requires Prox1 activity. Genes Dev 22: 3282–3291. 19056883 10.1101/gad.1727208 PMC2600759

[50] Aranguren XL, Beerens M, Coppiello G, Wiese C, Vandersmissen I, et al (2013) COUP-TFII orchestrates venous and lymphatic endothelial identity by homo- or hetero-dimerisation with PROX1. J Cell Sci 126: 1164–1175. 23345397 10.1242/jcs.116293

[51] Anderson SA, Baraban SC (2012) Cell Therapy Using GABAergic Neural Progenitors. In: Noebels JL, Avoli M, Rogawski MA, Olsen RW, Delgado-Escueta AV, editors. 'Jasper's Basic Mechanisms of the Epilepsies' 4th edition. Bethesda (MD): National Center for Biotechnology Information (US). 22787598

[52] Arber C, Li M (2013) Cortical interneurons from human pluripotent stem cells: prospects for neurological and psychiatric disease. Front Cell Neurosci 7: 10. 23493959 10.3389/fncel.2013.00010 PMC3595684

[53] Hunt RF, Girskis KM, Rubenstein JL, Alvarez-Buylla A, Baraban SC (2013) GABA progenitors grafted into the adult epileptic brain control seizures and abnormal behavior. Nat Neurosci 16: 692–697. 23644485 10.1038/nn.3392 PMC3665733

